# Revitalization of small millets for nutritional and food security by advanced genetics and genomics approaches

**DOI:** 10.3389/fgene.2022.1007552

**Published:** 2023-01-09

**Authors:** J. Lydia Pramitha, Jeeva Ganesan, Neethu Francis, Ravikesavan Rajasekharan, Jenita Thinakaran

**Affiliations:** ^1^ Karunya Institute of Technology and Sciences, Coimbatore, India; ^2^ Tamil Nadu Agricultural University, Coimbatore, India

**Keywords:** small millets, nutrition, therapeutic traits, ethnobotany, multi-omics

## Abstract

Small millets, also known as nutri-cereals, are smart foods that are expected to dominate food industries and diets to achieve nutritional security. Nutri-cereals are climate resilient and nutritious. Small millet-based foods are becoming popular in markets and are preferred for patients with celiac and diabetes. These crops once ruled as food and fodder but were pushed out of mainstream cultivation with shifts in dietary habits to staple crops during the green revolution. Nevertheless, small millets are rich in micronutrients and essential amino acids for regulatory activities. Hence, international and national organizations have recently aimed to restore these lost crops for their desirable traits. The major goal in reviving these crops is to boost the immune system of the upcoming generations to tackle emerging pandemics and disease infestations in crops. Earlier periods of civilization consumed these crops, which had a greater significance in ethnobotanical values. Along with nutrition, these crops also possess therapeutic traits and have shown vast medicinal use in tribal communities for the treatment of diseases like cancer, cardiovascular disease, and gastrointestinal issues. This review highlights the significance of small millets, their values in cultural heritage, and their prospects. Furthermore, this review dissects the nutritional and therapeutic traits of small millets for developing sustainable diets in near future.

## 1 Introduction

Recent changes in food habits related to multi-grains have generated enormous interest in food and nutritional security. People have now started to focus on the nutritional uptake of small millets and growing children are provided processed foods that meet the daily nutritional requirement (Muthamilarasan and Prasad, 2021). Small millets, otherwise known as “wonder cereals”, possess numerous health benefits and thrive in harsh conditions (Barretto et al., 2021). Increasing demand for nutrition has paved the way to revive these crops, which once ruled nations. The 11 small millet species—finger millet, foxtail millet, barnyard millet, little millet, proso millet, Kodo millet, fonio millet, teff, brown top millet, Job’s tears, and guinea millet—are commonly known as the lost crops of the world. They are so-called ‘small’ owing to their smaller seed size. Small millets are rich in micronutrients, essential amino acids, and vitamin B complex, which are very rare in our staple diets. Phytochemical studies in small millets have demonstrated their higher antioxidant contents and lower glycemic indexes compared to other food crops (Vetriventhan et al., 2020).

Several programs for diet schedules are now popularizing the use and consumption of small millets in various forms. This has resulted in the food processing industries producing value-added products like flakes, noodles, biscuits, cookies, batter, flour, bread, and rice analogs. Rice analogs are one of the most popular developments that increase the palatable value of small millets by heat extrusion (Zhang et al., 2020). Hence, the demand for small millets is increasing in food industries. In addition, small millets are free of gluten, and it is highly suitable for patients with celiac disease (Deshpande et al., 2015). Despite nutrition, traditional practices have emphasized their medicinal values. These crops were previously not only foods but were also used to treat diseases including cancer and snakebites. Hence, analyzing the therapeutic traits of small millets paves a new way for smarter food in near future. Their uptake in the regular diet would provide their caloric, nutritional, medicinal, and fiber rich-properties for a sustainable life (Banerjee and Maitra, 2020).

In response to emerging pandemics in the changing climatic scenario, crop scientists have focused on boosting the human immune system with natural supplements through food diversification. These efforts have necessitated the acceleration of small millet breeding programs (Upadhyaya and Vetriventhan, 2018). Small millet genomics has recently started analyzing agronomic traits. Dissecting the genes underlying nutritional and therapeutic traits by applying advanced omics will reveal novel metabolic pathways in cereals for biofortification (Kumar, and Kumar, 2015). Next-generation and third-generation crop sequencing and breeding have demonstrated the potential of genetic databases and tools to analyze the genomes of small millets (Aggarwal et al., 2022). Hence, the future relies on developing smart crops with climate resilience, higher nutrition, and therapeutic traits. Moreover, conserving the traditional landraces of small millets, their cultural heritage, and ethnobotanical values by national and international organization programs to protect the rights of tribal farmers is needed to preserve novel alleles for the future (Saha et al., 2016). The present review focuses on the significance of small millets, breeding techniques, and advanced approaches for improving their productivity by enhancing their desirable features in a sustainable cropping system.

## 2 Ethnobotanical significance and cultural heritage of small millets

**FIGURE 1:**
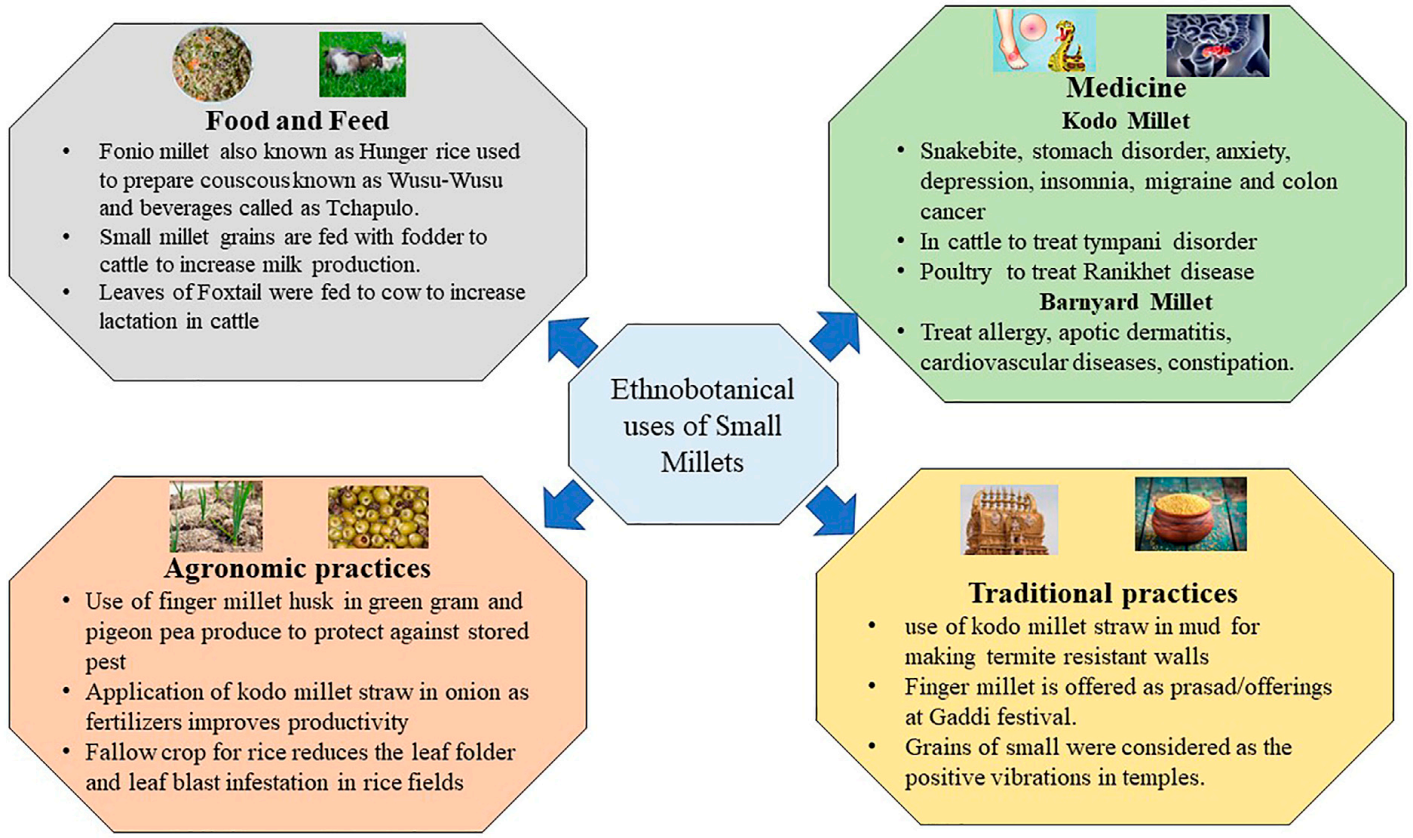
Ethnobotanical values of small millets.

Small millets have a profound significance in our cultural heritage and until now have played significant roles in temple festivals in tribal regions. These activities are preserved as traditional knowledge in the regulations put forth by PPVFRA, 2001 (Satyarthi et al., 2018). These traditions demonstrate that these grains were recognized by our ancestors for their nutraceutical and therapeutic values (Figure 1). One common practice in small grains was their presentation as a wedding gift to the bridegroom. The amount of grains gifted was treated as a prestige; the grains were also cooked, especially during puberty and childbirth celebrations (Rawat et al., 2021). Small millets are rich in folic acid; therefore, they were treated as a special entity for women to overcome anemic disorders. In Africa, fonio millet, commonly known as “hungry rice,” has similar importance. These grains are predominantly used to prepare couscous known as wusu-wusu. Fonio is best used in the preparation of beverages called *Tchapulo*, which is rich in minerals. Finger millet has a similar value in the processing of beer, and its malted products are often used in African tribal communities (Hitu et al., 1997). *Arake*, a distilled liquor, is prepared in Ethiopia with finger millet flour. Furthermore, people residing in Sudan predominantly consume a hot porridge of finger millet with banana or sugar juice, which is a staple dish in tribal zones. A sour bread known as *injera* is made from teff and is used in spicy stews by well-off tribal individuals. Teff has a unique role in Africa after fonio millet. Due to its cold tolerance in higher altitudes, it is popularly known as love grass. The novel features of these lost crops are also being conserved. The major morphotypes in fonio millet are Yoro, Ipordapia, Ipordawoun, Ipoagoa, and Iporni are conserved by communities including the *Hausa* and *pagans* in west African regions (Dansi et al., 2010; National Research Council, 1996).

Regarding the conservation of cultural heritage in India, the Malayali in Eastern Ghats continue to cultivate and conserve small millet landraces (Newmaster et al., 2013). The landraces of little, foxtail, and proso millets are conserved by the Kolli Hill tribes; the characteristics of these millets suggest the presence of novel alleles for future breeding programs (Venkatesan et al., 2015; Ragupathy et al., 2016). Tracking the records of the utilization of small millets in Chhattisgarh, India has revealed the use of Kodo millet straw in mud to make termite-resistant walls. The farmers of this region also used Kodo millet straw as fertilizers in onion fields to increase productivity. Another traditional practice was the application of finger millet husk in green gram and pigeon pea products to protect these grains from pest infestation during storage. The pot makers in Northern India also are using Kodo millet straw when baking pots. Moreover, the leaves of Kodo millet possess lecithin and are used for the treatment of snakebites, stomach disorders, and joint pain. In cattle, Kodo millet straw had a significant impact on treating tympani disorder. Additionally, the older grains of Kodo millet (3–4 years) were used to cure Ranikhet disease in poultry. In Africa and India, Kodo millet was a fallow crop after rice; in other rice fields, Kodo millet straw is usually spread in the fields to protect against leaf folder and blast (Rawat et al., 2021).

The small millet grains were also previously mixed with fodder to increase milk production in cows. Several recent agro-start-ups in India for cattle feed also practice this technique to enhance milk production in rural dairy farms (Bhat et al., 2018). In traditional practice, finger millet is often a prasad/offering in the Gaddi festival. This is believed to enhance the fruiting of non-flowering mango and tamarind trees. Furthermore, thick pastes of finger millet flour are used to treat fire burns, and these grains are considered to offer positive energy in temples (Sahu and Sharma, 2013).

In addition, barnyard millet is used to treat allergies, atopic dermatitis, cardiovascular diseases, constipation, and blood-related disorders. Moreover, Kodo millet is preferred for overcoming anxiety, depression, insomnia, migraine, and colon cancer. Foxtail millet is used to treat chicken pox, heart attack, fever, cholera, and gastric problems. The leaves of foxtail millet are also fed to cattle to increase lactation. These practices underscore the nutritional and therapeutic value of small millets in our heritage. The genetics underlying these traits could be explored and used to develop sustainable diets (Satyarthi et al., 2018).

## 3 Nutritional importance and quality parameters of small millets

**FIGURE 2:**
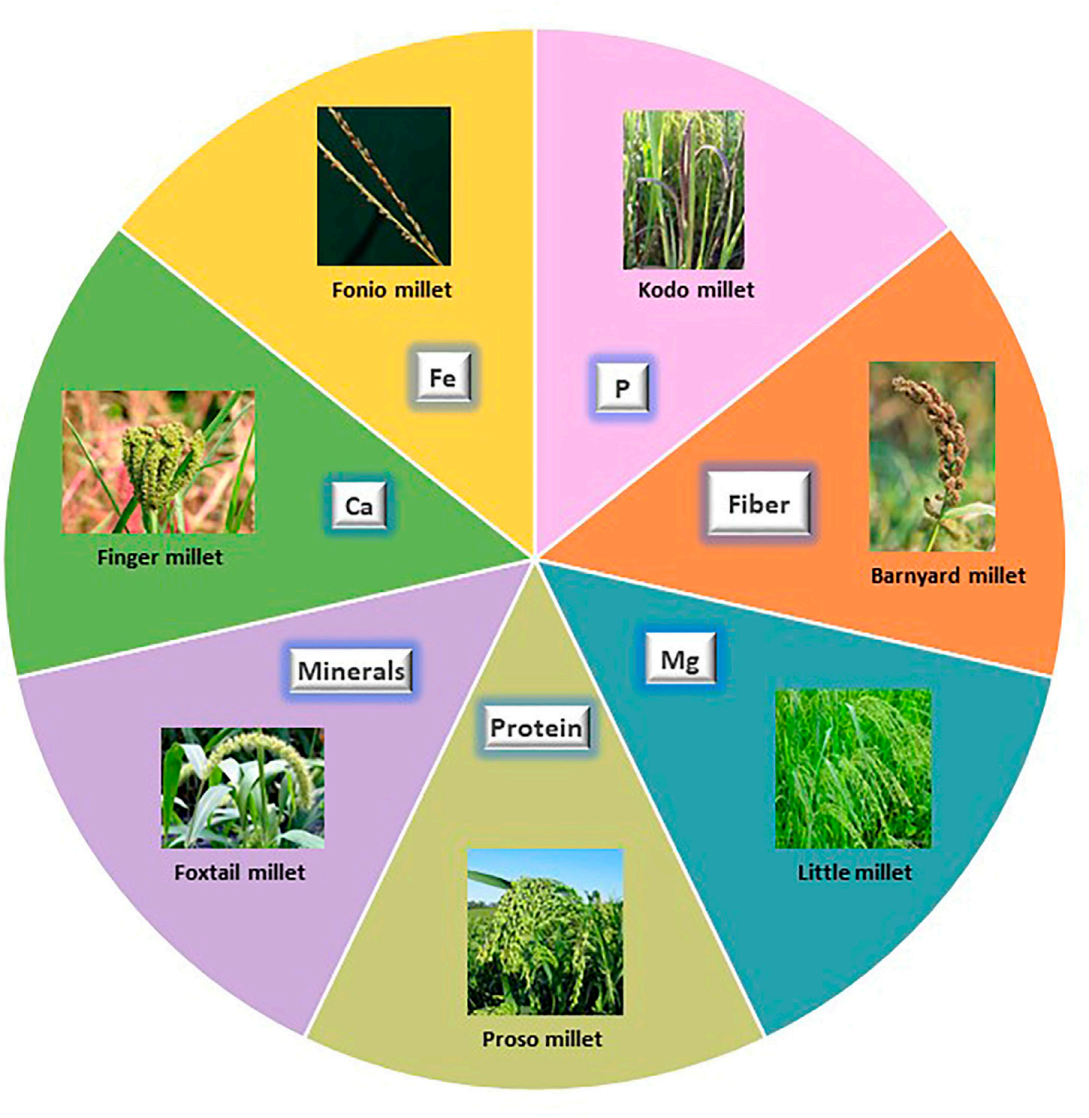
Nutritional uniqueness of small millets. Each sphere depicts the specialty of small millets in food.

Small millets are being considered for their paramount importance in nutritional aspects not provided by other staple crops. These species have profound nutritional benefits, especially their micronutrient and protein profiles (Pramitha Lydia et al., 2021). The 11 small millets species each represent a unique beneficial feature for dietary bowls (Figure 2). Considering their acceptability for consumption, they are highly preferred for patients with diabetes and celiac disease owing to their gluten-free and higher fiber content (Table 1). Our ancestors were involved in laborious work and these foods predominantly helped them to sustain healthy lives. Due to the comfortability provided by major staples in later generations, the importance of these crops was almost forgotten, and they lost their commercial value. However, some traditional farmers still preserve this cultural heritage through festivals and traditional practices. Arising pandemics and emerging unprecedented weather have caused us to reassess the nutritional benefits of these crops (Muthamilarasan and Prasad, 2021).

**TABLE 1:** Nutritional composition of small millets.

Crop	CHO (g)	Protein (g)	Fat (g)	Crude fiber (g)	Ash (g)	Ca (mg)	P (mg)	Fe (mg)	Zn (mg)	Mg (mg)
Finger millet	72.60	7.70	1.50	3.60	2.70	344.00	250.00	6.30	2.30	130.00
Foxtail millet	60.90	12.30	4.30	8.00	3.30	31.00	290.00	2.80	2.40	81.00
Proso millet	60.90	12.50	1.10	5.20	1.90	14.00	206.00	0.80	1.40	81.00
Barnyard millet	65.50	6.20	4.40	13.60	2.20	20.00	280.00	8.00	3.00	137.00
Little millet	65.60	10.40	1.30	7.60	1.30	16.10	220.00	1.30	3.70	133.00
Kodo millet	66.20	8.90	2.60	5.20	1.70	15.30	188.00	2.30	0.70	147.00
Rice	78.20	7.90	0.50	1.00	11.19	7.50	160.00	0.70	1.30	64.00
Wheat	64.00	10.60	1.50	2.00	0.94	41.00	306.00	5.30	2.70	138.00
Maize	18.70	3.27	1.35	2.00	4.83	10.00	89.00	0.52	0.46	37.00

Sources: Dey et al., 2022; Gopalan et al., 2009; Geervani and Eggum, 1989; Longvah et al., 2017.

Among the eleven species of small millets, finger millet and foxtail millet have larger areas of cultivation. Finger millet is known for its calcium, phosphorous, iron, and zinc content. It also has a higher caloric value due to its carbohydrate content. Foxtail millet is mostly preferred for its protein content, which is higher than wheat. It is also rich in minerals like phosphorous, iron, and zinc. Barnyard millet is known for its low glycemic index and high phosphorous and magnesium content. Among small millets, barnyard millet has the highest dietary fiber and niacin content (Pramitha Lydia et al., 2021). Kodo millet has the highest phosphorous content and radical scavenging activity owing to its high phenol content. Consuming Kodo millet reduces the risk of cardiovascular dysfunction (Rao Dayakar et al., 2017; Deepak Taggelli and Thakur, 2018). Little millet is known for its magnesium, phosphorous, and protein content. The unique feature of little millet is that it is rich in PUFA and flavonoids (Indirani and Devasena, 2021). Proso millet has a high protein profile following foxtail millet and also has higher magnesium and niacin content. Proso millet is also rich in essential amino acids like lysine, leucine, isoleucine, and methionine, unlike other major cereals (Pramitha Lydia et al., 2021). Fonio millet is preferably found in African regions and has high iron, dietary fiber, crude protein, flavonoid, GABA, and riboflavin content. It is highly nutritious owing to its high methionine and cysteine content. Teff is yet another wonder millet in Africa, which features the smallest grain and highest calcium and iron content. Teff is especially rich in lysine, which is deficient in all grains, and has comparatively higher protein and starch content. The other minor millets include brown top millet, Job’s tears, and guinea millet. These are also rich in phosphorous, iron, zinc, and vitamin B (Durairaj et al., 2019).

The demand for small millets is increasing in upcoming markets due to value-added products. Thus, their quality aspects should be considered for commercial production. Small millets are small-grained cereals and usually have a lower milling recovery than other grains. Therefore, uniformly sized, unbroken seeds are of concern in small millet production (Aggarwal et al., 2022). The colors vary from black to light yellow-colored grains, with consumers predominantly preferring light yellow- to brown-colored seeds for flour and cooking purposes (Durgad et al., 2021). Yellow-colored grains are also reportedly more aromatic compared to black grains (Aggarwal et al., 2022). Small millets are also a great source of resistant starch. The highest resistant starch values have been reported in Kodo millet, little millet, barnyard millet, and foxtail millet. Thus, these grains have nutraceutical value. As the grain yields of cereals deteriorate owing to harsh climates, small millets can be used to manufacture and process resistant starch in various industries (Kaimal et al., 2021). Rice analogs from small millet flours are also currently produced by heat extrusion and gelatinization. These rice granules are comparatively more desirable than normal rice varieties (Zhang et al., 2020).

Other quality parameters in small millet breeding include their fodder and forage value. Small millets are also used as fodder crops due to their higher biomass. Kodo millet, little millet, and proso millet are the most preferred animal feeds due to their higher palatability and crude protein content. Despite these factors, rancidity affects the storage of these flours in homes (Vetriventhan et al., 2020; Sruthi and Rao, 2021). Although the grains of small millets have a longer storage value, their flours are subject to rancidity; thus, efforts in advanced breeding techniques are needed to enhance the shelf life of small millet flours (He et al., 2015; Hariprasanna et al., 2014).

## 4 Breeding objectives and prospects in overcoming constraints

Small millets are among the less demanding crops in cultivation. They require minimum input from irrigation to fertilizers and pesticides. They can also thrive in harsh conditions, making them a reliable smart crop in the future (Durairaj et al., 2019). The major breeding objectives in small millets concern cultivation and post-harvest techniques. The initial objective predominantly focuses on higher yields. Yield is a complex trait that depends on numerous variables. The major factors causing yield loss in small millets are lodging and shattering. The increase of tillers in small millets with higher biomass at maturity results in lodging. Also, some crops are prone to shattering at harvest, which can be overcome with a stronger culm diameter while breeding for higher yield. Second, small millets have a non-preference in ideotype due to the spined hairy shoots and leaves. This causes challenges in managing field activities such as weeding and pesticide spraying. Hence, breeding for reduced bristles, spines in shoots, and leaves helps in proper crop management (Ganapathy and Patil, 2017).

Small millets are also less prone to diseases. Some of the major diseases infecting them are shown in Table 2. These infestations are observed only in endemic regions and conducive conditions caused by poor cultivation practices (Nagaraja and Das, 2016). Therefore, the crop protection schedule and its inputs in these crops are predominantly minimal (Ravikesavan et al., 2022). Finally, as a major objective in the post-harvest technique, smaller grain size causes poor milling recovery; thus, breeding for larger seeds would help minimize post-harvest losses (Table 3). Future efforts in small millet breeding should focus on improving the color, nutritional profile, fodder yield, flour quality, and reduced antinutritional traits (Vetriventhan and Upadhyaya, 2019).

**TABLE 2:** Major diseases affecting small millets.

S. No.	Millet	Disease	Causal agent
1	Finger millet	Blast	*Pyricularia grisea*
		Rust	*Puccinia substriata*
		Smut	*Melanopsichium eleusinis*
		Downy mildew	*Scleropthora macrospora*
		Seedling and leaf blight	*Drechlsera nodulosum*
		Cercospora leaf spot	*Cercospora eleusinis*
		Banded Blight	*Rhizoctonia solanii*
		Wilt or foot rot	*Sclerotium rolfsii*
		Bacterial leaf spot	*Xanthomonas eleusinae*
		Bacterial leaf blight	*Xanthomonas axonopodis pv. corocane*
		Bacterial leaf stripe	*Pseudomonas eleusinae*
		Ragi severe mosaic	*Sugarcane mosaic virus*
		Ragi mottle streak	*Ragi mottle streak virus*
		Ragi streak	*Maize streak virus*
	Foxtail millet	Blast	*Pyricularia setaria*
		Rust	*Uromyces-setaria italicae*
		Smut	*Ustilago crameri*
		Downy mildew	*Sclerospora graminicola*
		Udbatta	*Ephelis* sp.
		Bacterial leaf blight	*Pseudomonas avenae*
	Kodo millet	Head smut	*Sorosporium paspali*
		Rust	*Puccinia substriata*
		Udbatta	*Ephelis* sp.
		Kodua poisoning	*Aspergillus flavus/tamarii*
	Barnyard millet	Head Smut	*Ustilago crus-galli*
		Kernel smut	*Ustilago panici-frumentacei*
		Bacterial leaf blight	*Pseudomonas avenae*
	Proso millet	Head smut	*Sphacelotheca destruens*
		Bacterial leaf blight	*Pseudomonas avenae*
	Little millet	Rust	*Uromyces linearis*
	Teff	Rust	*Uromyces eragrostidis*
		Damping off	*Helminthospoirum poae*

Source: Nagaraja and Das, 2016.

**TABLE 3:** Specific breeding objectives for small millet resources.

S. No.	Small millet	Progenitor	Origin	Race	Breeding objective
1	Finger Millet (*Eleusine corocana* L.)	*Eleusine indica* x *Eleusine floccifolia/E. tristachya*	West Africa	1. Elongata a. Laxa	i) Higher yield, productivity, and non-lodging efficiency ii) Reduced tannins iii) Higher calcium and micronutrients iii) Bold seeded with higher finger length iv) Tolerant to drought, salinity, and diseases-like blast
				b. Reclusa	
				c. Sparsa	
				2. Plana a. Seriata	
				b. Confundere	
				c. Grandigluma	
				3. Compacta	
2	Barnyard millet				
	1. Japanese barnyard millet *(Echinochloa esculenta)*	*Echinochloa crus-galli*	Japan	1. Utilis	i) Higher yield and productivity ii) Bold seeded type iii) Higher quality and micronutrient profile
				2. Intermedia	
	2. Indian barnyard millet (*Echinochloa frumentacea*)	*Echinochloa colona*	India and Africa	1. Laxa	
				2. Robusta	
				3. Intermedia	
				4. Stolonifera	
3	Foxtail millet (*Setaria italica L*.)	*Setaria viridis*	China	1. Indica	i) Breeding for higher yield and productivity
				2. Maxima	ii) Higher protein, micronutrient status and palatability for fodder
				3. Moharia	iii) Breeding cultivars to resistance to pest and diseases
					iv) Varieties suitable for intercropping and early maturing
4	Little millet (*Panicum sumatrense* Roth. ex. Roem. and Schultz)	*Panicum psilopidium*	India and South East China	1. Nana	i) Bold seeded and higher productivity
				2. Robusta	ii) Higher protein and crude protein for enhanced fodder quality
					iii) Non-shattering and non-lodging type
					iv) early maturing, high fiber, and tolerant to shoot fly
5	Proso millet (*Panicum miliaceaum*)	*Panicum capillare* and *Panicum repens*	China	1. Miliaceaum	i) Suitable for intercropping and early maturing
				2. Patentissimum	ii) Higher productivity for grain and fodder
				3. Contactum	iii) Non-shattering and tolerant to biotic and abiotic stresses
				4. Compactum	
				5. Ovatum	
6	Kodo millet (*Paspalum scrobiculatum* L.)	*Paspalum sanguinale*	Africa	1. Regularis	i) Early maturing, non-lodging, and photosynthetic efficiency ii) Higher yield, productivity, bold seeded, and higher fodder quality iii) Cultivars for sole and mixed cropping iv) Tolerance to smut and other diseases
				2. Irregularis	
				3. Variabilis	

(Ravikesavan et al., 2022; Vetriventhan and Upadhyaya, 2018).

Although small millets have significant potential, the number of research programs remains low compared to other crops. This is due to the constraints involved in the smaller inflorescence and spikelets. These features restrict the possibility of attaining desirable recombinants. Recent approaches in hot water emasculation have resulted in the successful development of recombinants. Advances in mutation breeding with MutMap+ and genotyping facilities have allowed the dissection of novel alleles in small millets. Hence, there is a need for increased focus on small millet genetics and genomics using advanced omics approaches (Muthamilarasan and Prasad, 2015).

## 5 Progress of crop improvement in small millets

Initiatives on small millet improvement began in the early 1950s, when conventional breeding including pureline selection and pedigree breeding were ruling the varietal releases in all crops (Paroda and Mal, 1989). All small millets were highly self-pollinated and local collections by breeders were evaluated for line development. Varieties in small millets including CO 6 and CO (7) thenai in foxtail; CO (PV) 5 in proso millet; CO (samai) 4 in little millet; and CO 9, CO 13, CO 14, and Paiyur 2 in finger millet are examples of successful lines developed from recombination breeding. These varieties were released by Tamil Nadu Agricultural University by using standardized protocols for emasculation like hot water treatment and approaches (Ravikesavan et al., 2022). The millets were dominant in earlier civilizations; however, due to the palatability of major staples like rice and wheat, the small millets lost their presence among the population. Additionally, constraints like shattering, low yield, poor milling recovery, flour rancidity, and cooking time in small millets were major factors that drove these crops out of the staple source in the pipeline. Hence, breeder efforts were lost in the middle of the century after the green revolution (Vetriventhan et al., 2020).

Although these crops lost their economic value, few farmers relied entirely on this cropping pattern due to their adaptability in arid and harsh environments. Thus, these crops were always preserved as a cultural heritage among tribal populations and traditional farmers across generations. Overcoming the difficulties in crossing, mutation breeding in small millets later began to arise for varietal development. Mutation breeding in small millets started in the 1970s based on EMS and gamma rays. The frequency spectrum of macromutations with EMS and gamma in proso millet was studied by Ganapathy et al. (2008) and Bhave et al. (2016). Eswari et al. (2014) and Ambavane et al. (2015) also evaluated the effect of mutagenic frequencies with EMS and gamma rays in the previously released cultivars of finger millet. Foxtail mutation breeding experimented with different doses of EMS, DES, and gamma which also tended to affix the LD_50_ for isolating desirable mutants (Anittha and Mullainathan, 2018). Ramesh et al. (2019)assess the efficiencies of EMS and gamma rays in the CO (Kv) 2 strain of barnyard millet. Sood et al. (2020) evaluated the efficacies of different concentrations of sodium azide and gamma-ray treatments in barnyard millets. Thus, the LD_50_ values in small millets from earlier studies provided an understanding of the frequency of mutations induced in these genomes. Dosages of 500–600 Gy for finger millet and barnyard millet; 0.3%–0.45% EMS for finger millet, barnyard millet, and Kodo millet; 0.2% EMS for teff; 0.1 M nitrous acid in fonio millet for 4 h; and 0.03% nitrosoguanidine for finger millet induced desirable variations (Jency et al., 2016; Vetriventhan et al., 2020; Francis et al., 2022). Simultaneously, the genetic resources in small millet germplasm collections were also conserved as core collections in international research institutes. Due to efforts by Upadhyaya et al. (2016), the core collections from ICRISAT for little millet (460 accessions), foxtail millet (155 accessions), barnyard millet (89 accessions), and Kodo millet (75 accessions) were preserved and utilized. Other genetic reservoirs for small millets include NBPGR, the Ethiopian Biodiversity Institute, ICARDA, USDA, the N.I. Vavilov Russian Scientific Research Institute, the Ustymivika Experimental Research Station, and the Kenya Agricultural Livestock Research Station (Upadhyaya et al., 2008; Joshi et al., 2021).

The breeding of small millets has only recently accelerated due to increases in patients with diabetes and children with nutritional absorption issues. Moreover, the publication of the genome sequence of foxtail millet brought attention to the importance of these millets. Hence, relatively fewer molecular studies are published in small millets compared to the major staples. Still, almost all kinds of markers, from RFLP to AFLP, RAPD, EST-SSR, SSR, and SNPs have been utilized for marker-assisted selection. The first linkage map in small millets was developed with RFLP in foxtail millet; later, SSR and SNPs were used to map the QTLs in a high-density linkage map (Wang et al., 1998; Wang et al., 2017). Linkage maps with SNP markers were also later developed in proso millet by Rajput et al. (2016). A high-density linkage map with SNPs for finger millet was recently developed by Pendergast et al. (2022). Subsequently, molecular markers such as AFLP, RAPD, CAP, miRNA, EST, ISSR, SRAP, DEG, and SNP in proso millet were implied for genotyping the diversity, while EST, RAPD, and AFLP were used to analyze the calcium dynamics in finger millet (Habiyaremye et al., 2017). Fukunago et al. (2002b) and Desai et al. (2021) studied chloroplast and mitochondrial diversity in foxtail millet and barnyard millet, respectively. Among all molecular markers, SSRs were predominantly utilized in small millets and were also used in comparative genomics to analyze their lineages. Hence, a separate marker database for SSRs in foxtail was developed by Bonthala et al. (2014).

QTL mapping and trait mapping in small millets are in the initial stages; until now, QTL mapping for agronomic traits was performed (Table 4). The QTLs for traits like tiller number, branching, number of spikelets, bristles, panicle length and weight, plant height, grain weight/plant, and pericarp color have been identified in foxtail millet (Aggarwal et al., 2022). In finger millet, Babu (2014) and Ramakrishnan et al. (2016) identified QTLs for blast, neck blast, and leaf blast. Furthermore, in finger millet, the QTLs for P efficiency (Ramakrishnan et al., 2017), agronomic traits (Sharma et al., 2018), and biochemical traits like APX, CAT, GR, and SOD and POD activities (David et al., 2021) have also been studied. The populations mostly used for mapping in small millets included F2–F6 and RILs. The use of other mapping populations like NILs and double haploids remains to be explored (Vetriventhan et al., 2020; Zhang et al., 2021). Association mapping in foxtail millet and proso millet were predominantly studied with SSR and SNPs to determine the LD values for the diverse collections (Aggarwal et al., 2022; Boukail et al., 2021).

**TABLE 4:** Major QTLs detected in small millets.

S. No.	Crop	Trait	QTL	Chromosome	References
1	Foxtail millet	Tiller number	*Tdll*	5	Doust et al., 2004
		Axillary branching	*SQUAX 1*	6	
		Number of spikelets	*SPK*	9	
		Bristle number/primary branch	*BR*	8	
		Panicle length	*qPL 6.1*	6	Fang et al. (2016)
		Straw weight/plant	*qSWP 1.1*	1	
		Node number of the main stem	*qNMS 1.1*	1	
		Tiller number	*qTN 5*	5	Zhang et al. (2019)
		Plant height	*4115*	5	
		First main internode diameter	*qFMID 9.1*	9	Wang et al. (2017)
		Second main internode diameter	*qSM1D9.1*	9	
		Heading date	*qDTH2*	2	Yoshitsu et al. (2017)
			*qDTH7*	7	
		Plant height	*qPII5-2*	5	Wang et al. (2017)
		Panicle diameter	*qPD5-2*	5	
		Panicle weight	*qPW5-I*	5	
		Pericarp color	*qPC7-2*	7	
		Grain weight per plant	*qGWP3.3*	3	Liu et al. (2020)
		Straw weight per plant	*qSWP7.4*	7	
		Straw weight per plant	*qSWP9.1 *	9	
		Plant height	*qPH1.1, qPH1.2*	1	He et al. (2021)
			*qPH3.2*	3	
			*qPH5.1, qPH5.2*	5	
			*qPH6.3*	6	
			*qPH8*	8	
			*qPH9.1, qPH9.2, qPH9.4, qPH9.5*	9	
		Blast	*QLB-czas1*	1	Tian et al. (2021)
			*QLB-czas2*	2	
			*QLB-czas8*	8	
		Panicle length	*qPL9.5*	9	Zhi et al. (2021)
		Panicle diameter	*qPD9.2*	9	
2	Finger millet	Blast	*UGEP24*	3B	Babu (2014)
			*UGEP81*	6B	
		Neck blast	*UGEP18*	1B	
		Flowering date	*-*	1B	Pendergast IV et al. (2022)
		Plant height	*-*	3B	
		Panicle number	*-*	3B	
		Leaf blast severity	*-*	1B	
		Leaf sheath color	*-*	4B	Fukunaga et al. (2022a)
		Stb	*-*	2	
		DTH	*-*	2	
3	Proso millet	Lodging	*QLh.unac-lg5*	LG-5	Rajput et al. (2016)
		Peduncle length	*QPdl.unac-lg4*	LG-45	
		Grain shattering	*QGs.unac-lg5*	LG-5	
		100-grain weight	*QGw.unac-lg40*	LG-40	
		Grains per panicle	*QGpp.unac-lg4*	LG-1	
		Plant height	PH1.1	1	Liu C et al. (2022)
4	Teff	Days to heading	-	LG 17	Chanyalew et al. (2005)
		Days to maturity	-	LG 4	
		Grain yield	-	LG 6	Zeid et al. (2011)
		Panicle weight	-	LG 7	
		Panicle seed weight	-	LG 7	
		Panicle length	-	LG 7	
		Lodging index	-	LG 9	

Among small millets, foxtail millet is the most exploited crop in genomics analysis (Table 5). Thus, foxtail millet is a model crop to understand genes in other crops, owing to its amenable genome and crop duration. Hence, advanced omics, genome editing, and double haploid techniques have been standardized and utilized from foxtail millet to analyze the genomics in other small millets (Aggarwal et al., 2022). The first sequence of foxtail millet was completed by Bennetzen et al. (2012) and Zhang et al. (2012) with two different cultivars. An updated sequence of this millet was later released by Ni et al. (2017). Successively, the finger millet whole genome sequence was published by Hittlamani et al. (2017) and Hatakeyama et al. (2018). The genome of sequences of barnyard millet and proso millet were also completed and published in China by Guo et al. (2017) and Zou et al. (2019), respectively. The genome sequences of teff (VanBuren et al., 2020) and fonio millet were recently completed (Abrouk et al., 2020). In addition, the protocols for genetic transformation with agrobacterium and biolistic approaches in finger millet, agrobacterium and biolistic methods in foxtail millet, biolistic methods in barnyard millet, agrobacterium-mediated approaches in teff and fonio, and agrobacterium-mediated methods in Kodo millet are now available (Vetriventhan et al., 2020; Bhatt et al., 2021). These advances are expected to enhance the prospects of understanding the underlying genomics of small millets. However, there remain research gaps in small millets regarding key nutritional and medicinal traits that could be used to revitalize our diets.

**TABLE 5:** Major omics approaches conducted in small millets.

S. No.	Crop	Omics approach	Key finding	Reference
1	Foxtail millet	Genomics	Reference Genome sequence of “Yugu1” genotype. Genome size was predicted to be 510 Mb	Bennetzen et al. (2012)
		Genomics	Draft genome sequence of “Zhang gu” genotype. Estimated genome size was 423 Mb	Zhang et al. (2012)
		Transcriptomics and metabolomics	Phenylpropanoid, flavonoid, and lignin biosynthesis pathways, and lysophospholipids plays an important role in salinity tolerance	Pan et al. (2020)
		Metabolomics	Metabolite profiling of seeds. Region-specific differential expression of 20 metabolites	Yang et al., 2021
		Transcriptomics and metabolomics	Identified vital genes involved in carotenoid metabolism and regulation	Li et al. (2022)
2	Finger millet	Genomics	Draft genome sequence of “ML-365” genotype	Hittalmani et al. (2017)
		Genomics	Draft genome sequence of “PR202” genotype. Estimated genome size was 1.5 GB	
				Hatakeyama et al. (2018)
		Integrated transcriptomics and proteomics	Differentially expressed genes (DEGs) associated with corresponding differentially expressed proteins (DEPs) involved in drought tolerance were identified. They were enhanced in hydrolase activity, glycosyl bond formation, oxidoreductase activity, carbohydrate binding and biosynthesis of unsaturated fatty acids	Li W et al. (2021)
		Multiomics	Fifteen putative genes involved in Fe and Zn homeostasis pathways were identified	
			Function annotation of the genes identified high similarity with rice, wheat, maize, barley, and foxtail millet.	Chandra et al. (2020)
		Transcriptomics and metabolomics	Supplementation of silica to osmotic-stressed plants reprograms fatty acid biosynthesis to impart tolerance	Mundada et al. (2021)
3	Proso millet	Genomics	Draft genome sequence of ‘Longmi4’ genotype. Estimated genome size was 887.8 Mb	Shi et al. (2019)
		Genomics	Draft genome sequence of a landrace from Northern China (accession number 00000390). Estimated genome size was 923 Mb	Zou et al. (2019)
		Transcriptomics	Low N-tolerant genotype had higher efficiency of N uptake and utilization and photosynthesis of leaves	Liu T et al. (2022)
		Transcriptomics	Faster recovery of the photosynthetic genes, ROS scavenging system transcriptional responses, and regulation of jasmonic acid signal transduction pathway played a critical role in the drought-tolerant genotype ‘Neimi 5’	Zhang et al. (2019)
		Metabolomics	Grain metabolite and phenolic profiling in Korean varieties	Kim et al. (2013)
		Metabolomics	No significant differences were observed between metabolites of millets grown conventionally and organically	Liang et al. (2018)
		Metabolomics	Hundred metabolites were differentially expressed between colored grain and white grain type. Identified metabolites involved responsible for antioxidant and quality characters in the seed	Li J et al. (2021)
4	Barnyard millet	Genomics	Draft genome sequence of ‘STB08’	Guo L et al. (2017)
		Transcriptomics	Differentially expressed genes and regulatory mechanisms involved in drought tolerance and Fe and Zn accumulation	Jayakodi et al. (2019)
		Metabolomics	Metabolomic profiling of contrasting genotypes of Fe content identified differentially expressed metabolites at different stages of spike development	Padhiyar et al. (2022)
5	Teff	Genomics	Draft genome sequence of “Tsedey (DZ-Cr-37)”	Cannarozzi et al. (2014)
		Transcriptomics	Annotation of 3800 transcripts	Cannarozzi et al. (2014)
		Comparative genomics	Synteny with sorghum	Cannarozzi et al. (2014)
6	Little millet	Metabolomics	Twenty-five metabolic pathways were impacted by drought stress	Dhawale (2022)
		Transcriptomics	SSR marker identification and functional annotation of unigenes	Desai et al. (2021)
		Transcriptomics	Molecular mechanism and DEGs under drought and saline stress	Das et al. (2020)
7	Kodo millet	Transcriptomics	Identification of differentially expressed genes and molecular pathways involved in dehydration stress	Suresh et al. (2022)

## 6 Genomics of climate resilience in small millets

The average global temperature is predicted to rise by 4–5°C by the end of the twenty-first century, which will adversely affect the growth of cereals crops like wheat and rice. Small millet crops, apart from being nutritionally superior to major cereal crops, are inherently tolerant to abiotic stresses like drought, high temperature, cold, poor soil fertility, and salinity (Goron and Raizada, 2015; Singh et al., 2021). They are physiologically sustainable under adverse environmental conditions owing to their excellent and efficient water and nitrogen use (Baltensperger, 2002; Saha et al., 2016). These characteristics highlight small millets as ideal smart crops for cultivation in the context of climate change. They can also aid in creating climate resilience in major cereals like wheat and rice (Bandyopadhyay et al., 2017). Hence, understanding the genetic and molecular mechanisms controlling stress tolerance in millets is critical. Modern and conventional breeding should work together to expedite the dissection and utilization of these complex mechanisms.

The advent of next-generation sequencing technologies (NGS) has revolutionized the field of genomics and created significant molecular information. Although the benefits of this technology were delayed in small millets due to limited funding and research, the genome sequences of foxtail millet, finger millet, proso millet, teff, Japanese barnyard millet, and white fonio are now available (Zou et al., 2019; Vetriventhan et al., 2020; Wang et al., 2021). Whole-genome sequencing and annotation of the ML-365 finger millet genome revealed TFs (transcription factors) and genes related to drought tolerance and the C_4_ photosynthetic pathway. A total of 2,866 drought-responsive genes were associated with WRKY, MYB, MYC, ZFHD, NAC, ABF, AREB, GRF, and NF-Y transcription factors (Hittalmani et al., 2017). In proso millet, 180 *NAC* TFs were identified and differentially expressed under various drought treatments. The expression levels of 31% of *PmNAC* (Proso millet *NAC*) genes were upregulated in the roots, indicating the critical role of root characteristics in drought tolerance (Shan et al., 2020). Numerous enzymes, including signal recognition particle receptor, farnesyl pyrophosphate synthase, calcineurin B-like interacting protein kinase 31, serine–threonine protein phosphatase 2A, and others were activated by drought stress in finger millet. Several housekeeping and basal regulatory genes were also activated by drought. Novel drought-associated genes reported in the crop included pentatricopeptide repeat proteins and tetratricopeptide repeat proteins (Parvathi et al., 2019).

Salinity-tolerant finger millet genotypes show upregulation of many genes governing cell growth and differentiation. In the salinity-tolerant strain Trichy 1, genes involved in flavonoid biosynthesis were selectively down-regulated (Rahman et al., 2014). Transcriptomic and metabolomic analyses of contrasting genotypes for salinity tolerance showed that lysophospholipids, phenylpropanoid, flavonoid, and lignin biosynthesis pathways were crucial in the salinity tolerance of foxtail millet. The tolerant Yugu2 strain showed increased antioxidant enzyme activity and non-enzymatic antioxidant content (Pan et al., 2020; Puranik et al., 2011). The genome-wide gene expression profile in foxtail millet showed that most of the crop’s drought-responsive genes were associated with photosynthesis, signal transduction, and TFs (Qin et al., 2020). Apart from the well-known stress tolerance-associated genes like peroxidases and glutamine synthetase, leaf tissue-specific expression of ricin-B lectin-like was reported in little millet under salt and drought stress. Alcohol dehydrogenase was also upregulated in both root and leaf tissues of the crop under both stresses (Das et al., 2020). Photosynthesis was a key factor in the ability of Indian barnyard millet to adapt to dry conditions, as shown in a comparative transcriptome analysis between the plant and its wild relative, barnyard grass (*Echinochloa crus*-*galli*) (Jayakodi et al., 2019). Thus, the unique expression for climate resilience in small millets suggests novel alleles for use in stress breeding programs (Saha et al., 2016).

## 7 Innovative breeding methods to improve nutrition and stress tolerance

Innovative breeding methods can be used to speed breeding progress and reveal genes responsible for stress tolerance and nutritional quality improvement in small millets. Additionally, this information can be used to enhance these traits in susceptible major cereal crops. Whole-genome sequencing has revolutionized the breeding and biotechnological approaches in crop improvement. The genome sequences of small millets have generated genomic resources that can be used to identify the gene position, selection, and introgression of desirable traits into other varieties, species, or crops. By considering the genome sequence of a single individual as a reference genome, we may miss many key variations present in crop genetic diversity. This reference bias can be overcome by the concept of a pangenome, wherein several individuals of the species are sequenced to better represent the crop’s diversity. This method can distinguish the diversity as core (shared among different genotypes of the species) and variable (may be absent in some individuals) genome (Tettelin et al., 2005; Morgante et al., 2007). In small millets, where genome sequence information is limited, the sequences of panicoid species can be combined using pangenome and comparative genome approaches to improve breeding efficiency (Bennetzen and Freeling, 1997; Sharma et al., 2018; Tay Fernandez et al., 2022). Moreover, trait-based pangenomes can also be devised for stress tolerance and nutritional quality (Tay Fernandez et al., 2022).

Comparative genomics between C_4_ and C_3_ grasses provides a better understanding of their evolutionary paths to accelerate the research goal of improving the photosynthetic efficiency and drought tolerance in common staple crops (Chapman et al., 2022). High-throughput sequencing platforms have made the identification of single-nucleotide polymorphisms (SNPs) easier. Currently, SNPs are the main genetic variations or markers used in marker-assisted selection (Lata and Prasad, 2013). Genome-wide association studies (GWAS) and nested association mapping (NAM) can be used to establish marker–trait associations and detect powerful QTLs. NAM uses multiple mapping populations, has higher recombination events, and can detect tightly associated QTLs compared to traditional QTL mapping. Though this approach is widely used to identify candidate genes in major cereal crops, it has not yet been applied in underutilized crops like small millets (McMullen et al., 2009; Bouchet et al., 2017).

Mutation breeding has evolved from conventional methods to MutMap populations and targeted mutagenesis. MutMap and MutMap + are genome sequence-based mutation breeding approaches that facilitate rapid gene identification and isolation. The MutMap method has been used to map the dwarfing gene *D3* on chromosome 8 of the foxtail millet genome. The dwarf mutant also shows reduced drought tolerance, indicating critical trait associations (Fan et al., 2017). Mutmap-based cloning was used to establish the regulatory role of the WRKY transcription factor in panicle and seed development. MutMap + does not require crossing between mutant and wild type and is a desirable approach in millet crops in which crossing is tedious (Fekih et al., 2013). New plant breeding techniques like CRISPR-Cas9, TALENs, ZFNs, mega nucleases, oligonucleotide-directed mutagenesis (ODM), cis-genesis, transgenesis, and RNA-dependent methylation (RDM) can also be explored for small millet crop improvement (Schaart et al., 2016). These techniques can be used to decrease anti-nutritional content like phytates, polyphenols, and tannins. The higher levels of proteases and amylose inhibitors that impede millet digestibility can also be reduced using site-targeted modifications (Vinoth and Ravindhran, 2017). In foxtail millet, bioinformatic database information was used to identify target genes for waterlogging tolerance in designing guide RNA for clustered regularly interspaced short palindromic repeat (CRISPR)-aided activation of tolerance-associated transcripts (Abdulla et al., 2021).

Various approaches exist for the transgenic biofortification of finger millet and other crops. Increasing the calcium (Ca^2+^) storage capacity in edible parts by modifying the expression of transporter proteins, including the overexpression of channel proteins and Ca^2+^-binding proteins to increase calcium accumulation and mutagenesis-induced alterations of calcium content (Wyatt et al., 2002; Morris et al., 2008; Conn and Gilliham, 2010; Sharma et al., 2018). Other modern techniques like double haploid production and reverse breeding in millet crops may be useful for fixing genetically desirable cross combinations (Gis et al., 2019). Creating such platforms in the model crop foxtail millet may provide opportunities to dissect complex C4 pathways in millets and exploit their role in stress tolerance (Jacquier et al., 2020). Cheng et al. (2021) reported haploid embryo induction through the CRISPR-Cas9-mediated knockout of *SiMTL* in foxtail, which is an ortholog of MATRILINEAL/NOT-LIKE-DAD/PHOSPHOLIPASE A (*MTL/NLD/ZmPLA*) in maize used for haploid induction (Gilles et al., 2017; Kleter et al., 2019).

Transcriptomics is yet another method that has generated enormous genomic information even in crops in which genome sequence data are not available. Transcriptome sequencing (RNA-Seq) reveals genome-wide information on functional genes, differential expression of these genes, and their regulatory mechanisms. This method is widely used as it is cheaper than building a genome assembly and reveals the transcriptional activity based on the time and location of the observation. It can also provide insights into various metabolic pathways of the crop (Guo et al., 2021). Metabolomics, another promising omics method, was used to profile metabolites associated with drought stress response in little millet (Dhawale. 2022). The integrative use of transcriptomics and metabolomics was applied to identify key pathways involved in salinity tolerance in foxtail millet (Pan et al., 2020). Metabolomics analysis revealed the environmental and geographical influences on the bioactive nutrient profile of foxtail millet and the key biochemical pathways (Yang et al., 2021). Thus, combining advanced techniques to understand the genetic regulation of crops provides new gateways for the development of desirable plant types.

## 8 Advanced omics approaches in small millets for trait-specific breeding

**FIGURE 3:**
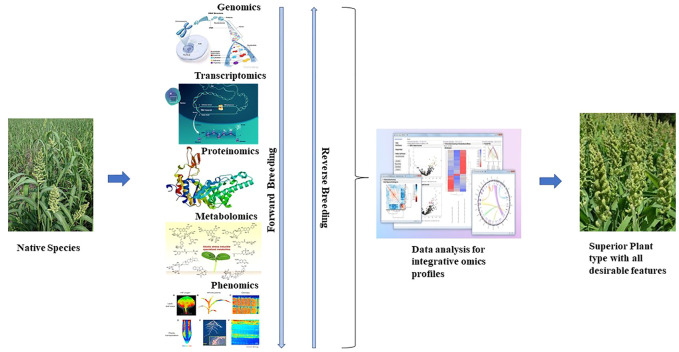
Flow of multi-omics in rapid breeding programs.

Recent advances in next-generation sequencing have applied multiple omics. Integrated omics is a rapid platform to hasten the selection process in plant breeding programs (Figure 3). Genomics plays roles from locating QTLs to developing trait-associated markers. Structural genomics has been utilized to locate the trait of interest in genetic and physical maps. Furthermore, positional cloning of QTLs by advanced QTL-seq approach has also allowed the identification of the exact location and sequence of candidate genes, paving the way for the design of allele-specific markers (Fan et al., 2017). Inclusively, genotype by sequencing has more precisely revealed the population structure of diverse collections, which has enabled the detection of novel alleles. Allele-specific markers are now used in plant breeding programs and are more reliable than the conventional markers used previously (Scossa et al., 2021).

Functional genomics also plays a major role in identifying the functions of genes relative to the trait of interest. Mutagenomics, epigenomics, and pangenome analysis are involved in depicting gene functions. Metabolomics, proteomics, trancriptomics, ionomics, and phenomics are also used in plants to dissect functional roles in gene expression. However, to understand the complete biological process of an organism, multi-omics is essential and also plays a role in the rapid breeding of crops (Kumar et al., 2021). Previously, metabolomics and transcriptomics were associated with plant phenotypes of major traits (Table 5). Recently, owing to advances in omics technology, an integrated approach has been implemented in crops. GWAS combined with metabolite profiling has been used to detect biochemical and genetic processes in major staples (Luo, 2015; Matsuda et al., 2015). Combined HRPF and GWAS was used to inform biomass and yield in rice (Yang et al., 2015); photosynthesis and growth rates in maize were analyzed by the combination of metabolomics and ionomics (Guo R et al., 2017); metabolomics, ionomics, and genomics involving QTL mapping were applied to evaluate iron and zinc in rice (Pinson et al., 2015); integrated mutagenomics and phenomics were used to understand increased lycopene in tomato (Li et al., 2018); mQTL analysis and mGWAS have been applied in maize and rice (Li K. et al., 2019; Tiozon, 2021), and combined transcriptome and metabolome study have been used to understand defense response in wheat *Trichoderma harzianum* strain T22 (Coppola et al., 2019) to detect key metabolites and candidate genes involved in the pathways for grain yield, secondary metabolites, and disease resistance (Yang et al., 2021).

Finally, functional genomics plays a major role in hybrid performance. The prediction of genotype performance by multi-omics is more precise than the conventional techniques (Yang et al., 2021). Future goals of integrated omics include the development of a proteome and metabolome atlas depicting the major genes in metabolic pathways, which also ensures the development of advanced databases for integrated big data in molecular breeding (Peng et al., 2020). These future innovations could frame a new way to design crops for favorable traits by *de novo* domestication and allow the rapid development of crop ideotypes (Choi, 2019).

## 9 Therapeutic value and its incorporation through genomics-assisted breeding techniques

Small millets are known for their nutritional value; however, understanding their significance in ethnobotany reveals their therapeutic value. The wonder grains of small millets are remembered as medicines for cures; thus, studying these values offers a greater scope for feeding people with a healthier diet. Each small millet has a potential medicinal value; for example, barnyard millet is known for its fiber content and thus is highly recommended to patients with heart disease and diabetes mellitus. Barnyard millet also has a higher lysine and iso-leucine for blood formation and lipid metabolism in organisms. The components of barnyard millet have the highest linoleic acid, which is a major explanation for the plant’s antioxidant and immunological activities. The phytochemicals in barnyard millet include alkaloids, tannins, terpenoids, and flavonoids (Renganathan et al., 2020). Moreover, finger millet is rich in tryptophan; therefore, it is prescribed for decreasing appetite in weight loss programs. Finger millet also includes threonine, which obstructs the formation of fat in the liver and regulates cholesterol levels. Due to its higher calcium, potassium, and iron levels, this millet is also suggested for use in growing children, lactating women, and anemic people (Verma et al., 2018). Ragi is rich in phytates, tannins, and trypsin inhibitors. Proso millet is predominantly known for its highest protein content and is prescribed as a potential therapeutic agent for diabetes mellitus II. This is combined with folic acid, vitamin B6, phenolic acid, ferulic acid, chlorogenic acid, syringic acid, and caffeic acid. Proso millet has shown effectiveness in curing heart disease and preventing breast cancer (Goron and Raizada, 2015). However, foxtail millet has higher linoleic acid, tocopherol, phenol, and flavonoid levels, which enhance its therapeutic value. Additionally, it supplies copper for regulated metabolic reactions. Moreover, small millets contain flavonoids and folic acid and are often used to enhance immune function. Finally, fonio millet is a good source of GABA and folic acid and has the highest iron content (Satyarthi et al., 2018).

These small millet components have been associated with health. Research on their therapeutic value and their stability in the grains is needed and could be a foundation for improving the medicinal values of major staples. The biofortification of grains with micronutrients is reaching new heights and incorporating flavonoids, folates, and GABA in mainstream foods to strengthen our immune response to overcome new emerging diseases. Although rice landraces are used as sources of folate, GABA, and flavonoids, their expression in processed grains is a concern. Whole brown rice and pigmented rice are highly nutritious; however, low percentages of people have access to these landraces. Hence, small millets could be an efficient source for enhancing the medicinal value of food, and its genomics may reveal the expression of these traits in processed foods (Tiozon 2021).

Thus, studies are needed for the identification of QTLs for these traits from mapping populations; association studies in small millets to identify factors related to micronutrients, therapeutic traits, and yield; metabolite genome-wide association studies; functional characterization of key genes; dissection of stable donors; and candidate gene analysis (Xia et al., 2021). Correlation and association studies on medicinal and yield values would help improve multiple traits in small millets (Tiozon 2021). Linkage maps for agronomic traits are currently available in foxtail millet and must be further improved to include micronutrient and medicinal traits. Since small millets are climate-resilient, achieving overall improvements in yields, micronutrients, and therapeutic profiles will be rewarding for smart foods (Aggarwal et al., 2022).

## 10 Road map for enhancing nutritional security with small millets

The current status in crop production sheds light on enhancing small millets productivity, which is influenced by many factors. The priority is to increase the areas under small millet cultivation. Until now, the cultivation of small millets was restricted to marginal and rainfed farmers who use these as cover crops to conserve arable lands in harsh conditions (Merga, 2018). These farmers also have poor access to high-quality seeds for sowing and the availability of pure small millet varietal seeds must be meticulously formulated from seed production plots. Second, increasing the cultivation area requires accelerating the market demand. Market consumers must be familiarized with diversifying their food habits for a sustainable life (Ravikesavan et al., 2022). Food diversification results in crop diversification and the potential to reintroduce lost and underutilized crops to mainstream cultivation. Recent cropping systems have also been altered to include small millets and they are now incorporated as an intercrop with legumes for a higher profit (Maitra, 2020). This positively impacts our ecosystem for a balanced chain and may also minimize genetic erosion (Deshpande et al., 2015).

Moreover, public communities have recently focused on physical fitness, with trainers in urban regions counseling their trainees on food habits. These efforts involve nutritional supplements and popular advertisements for artificial health supplements have appeared in diet schedules. These schedules could soon be replaced with small millet-based supplements. Small millets are rich in folic acid, flavonoids, terpenoids, resistant starch, and other phytochemicals that regulate cellular metabolic activities. Hence, in-depth research on the therapeutic value of small millets and their enrichment could replace artificial supplements in fitness programs (Durairaj et al., 2019; Lloyd and Kossmann, 2021).

**FIGURE 4:**
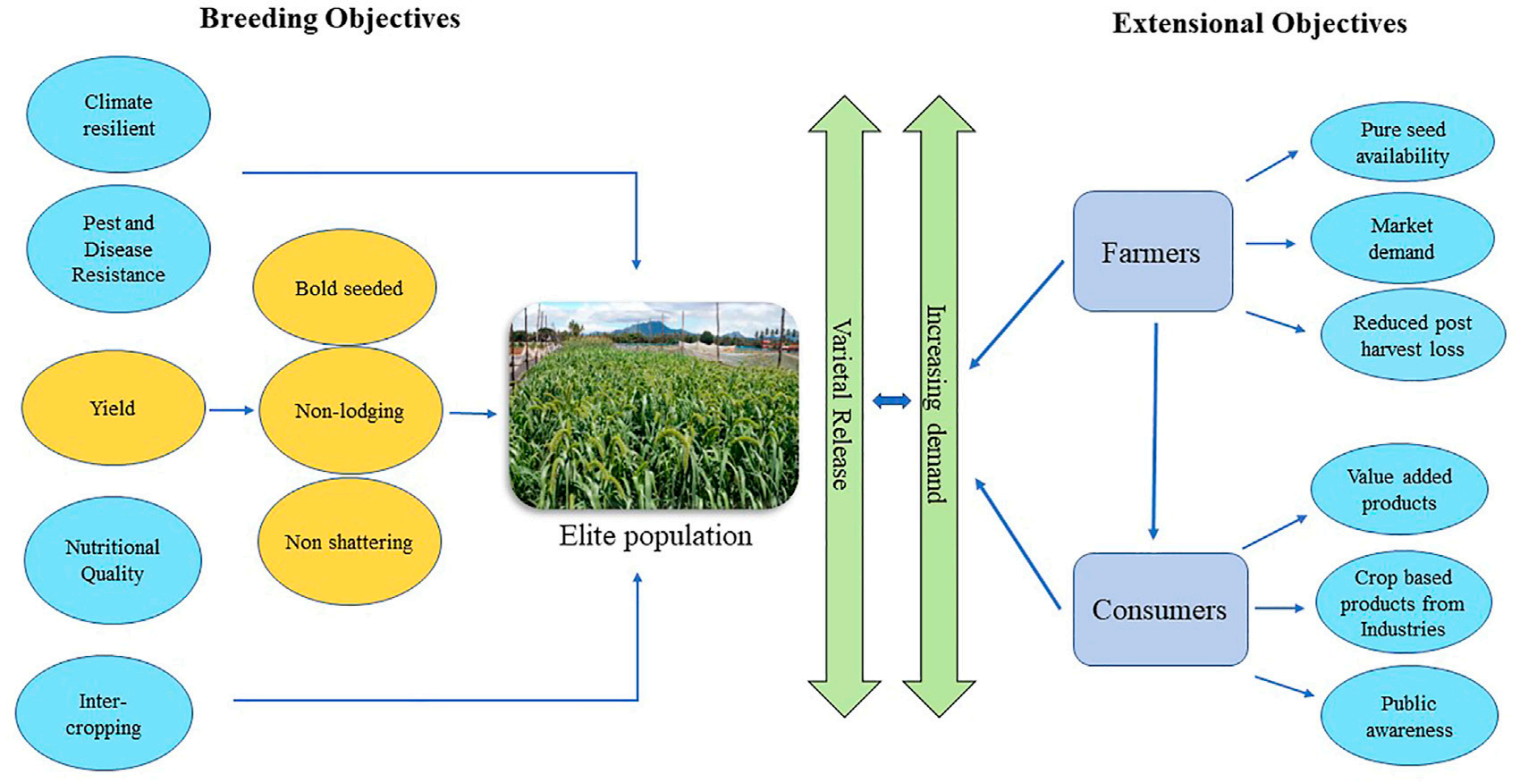
Road map for revitalizing small millets.

Another major stream in small millets that requires consideration is the incorporation of breeding strategies to yield desirable traits. Weedy features in small millets need improvement through selection. The characteristic features like shattering, lodging, small seeds, spined shoots, bristles, and awns must also be considered in the development of crop ideotypes in small millet breeding (Banerjee and Maitra, 2020). Further breeding objectives for analyzing their nutritional stability, bioavailability in processed foods, multigrain products, phytochemical expression, uniform maturity, fertilizer responsiveness, and biotic stress tolerance must be emphasized to provide higher yield and productivity (Figure 4). Thus, small millets could be an alternative source of nutritional and therapeutic traits in our regular diet (Adekunle et al., 2018; Upadhyaya and Vetriventhan, 2018). Food processing industries are also now moving toward small millets for the extraction of resistant starch, which opens a new market for small millet demand. In response, countries have increased their focus on small millets (Kaimal et al., 2021).

Small millets are arising as major cereals in countries like Ethiopia where other crops strive hard to feed the populations. The staple crop of Ethiopia is teff and Europe and the US are increasing their production of this grain owing to its nutritional value (Woldeyohannes et al., 2022). Proso millet is yet another “poor man’s food”. The cultivation of proso millet is increasing in the northern US and China. From a catch crop, it is now evolving as an alternative to the major food basket across countries (Bhat et al., 2019). Foxtail millet is well renowned for its grain and fodder. Recent ventures in foxtail yield and productivity are welcomed by farmers in Telangana and Andhra Pradesh in India. Internationally, foxtail millet is also favored for its various savories and recipes in China, Europe, and the US. Millet is also being explored as a biofuel, which has generated large industrial interest (Petti et al., 2013; Aggarwal et al., 2022). In addition, Western Africa is also now considering the fonio millet value chain in its market as a future for crop and food diversification based on small millets (Kanlindogbe et al., 2020; Ibrahim Bio Yerima and Yerima, 2021). The new-age policies from the government for introducing finger millet in Nepal and enhancing its cultivation in Ethiopia (Gebreyohannes et al., 2021) provide a new gateway for future diets. Recently, a global strategy for the intake and conservation of small millets proposed by Bramel et al. (2022) also enforces the need to enhance the cultivation of small millets to meet the continuously increasing demand. As discussed in the previous works on small millets, these crops can thrive to address future food and nutritional hunger (Muthamilarasan and Prasad, 2021; Renganathan et al., 2020).

## 11 Conclusion

This review highlights the overall value of small millets in our daily lives. Small millets were important food crops in ancient civilizations. Due to shifts in human dietary habits, these crops are now underutilized as food. By analyzing recent progress toward the use of small millets, we can realize support from government-aided projects and international and national collaborations to revitalize the genetic resources in small millets. Insights into the novel traits in small millets have demonstrated their paramount importance in nutritional and climate resilience. Therefore, this states the reasons behind the reverence of these crops in the cultural heritage of our ancestors. From these ethnobotanical records, we can conclude that breeding programs for improved varieties will restore the importance of on-farm conservation of wild and traditional landraces of small millets.

Small millets are climate-resilient crops that can meet the need for food and fodder and act as nutritional supplements. Hence, they are well considered as nutri-cereals and smart crops. Our detailed survey of studies in small millets revealed their great scope for applying advanced omics techniques to tap the genetic reasons for climate resilience and nutrition. Among small millets, the prospects of research in foxtail are higher and it has been utilized as a model crop system. Thus, small millets could act as a model crop for novel genes and mechanisms for manipulation by comparative genomics in mainstream cereals for increased stress tolerance and therapeutic traits. In this context, recent trends in food habits among the public have also begun to shift toward small millets, as evidenced by multigrain value-added products, processed foods, and new recipes in the markets. Therefore, small millets could be a major crop in future and a component in diversifying our food habits for a healthier life.
